# A Novel Triple Combination To Combat Serious Infections with Carbapenem-Resistant Acinetobacter baumannii in a Mouse Pneumonia Model

**DOI:** 10.1128/spectrum.02710-21

**Published:** 2022-08-17

**Authors:** Lamiaa A. Al-Madboly

**Affiliations:** a Department of Pharmaceutical Microbiology, Faculty of Pharmacy, Tanta University, Tanta, Egypt; Labcorp

**Keywords:** *A. baumannii*, synergism, triple combination, carbapenemases, pneumonia

## Abstract

The ongoing crisis of antimicrobial resistance demands novel combinations between antimicrobials and nonantimicrobials to manage infections caused by highly resistant pathogens. This study aimed to evaluate the effect of combining sodium ascorbate and/or apo-transferrin with imipenem, forming double and triple combinations, against 20 multiple-carbapenemase-producing Acinetobacter baumannii strains using the checkerboard test, time-kill assay, and disc diffusion test. The results of the checkerboard assay revealed that all double combinations showed indifference, while only triple combination recorded a synergistic effect (fractional inhibitory concentration index [FICI] < 0.8) in 95% the test isolates. Moreover, the MIC of imipenem (MIC_imp_) was strongly reduced (up to 128-fold reduction) after treatment with the triple combination against highly resistant isolates and reached the susceptible range. The time-kill assay revealed that the triple combination led to a 4-log_10_ reduction in the CFU at 8 h compared with the initial bacterial count, and no viable count was recorded at 10 h. The mouse pneumonia model showed restoration of lung function and structure, with mild to moderate residual inflammation and moderately congested vessels observed 8 h following administration of the triple rescue therapy. Additionally, normal lungs with normal patent alveoli were detected 72 h following treatment. Accordingly, sodium ascorbate and apo-transferrin are promising adjunct biological agents with the potential to restore the effectiveness of critically essential antibiotics like imipenem, commonly used for the treatment of A. baumannii infections.

**IMPORTANCE** Combination therapy provides a perspective to threat multidrug-resistant (MDR) strains. The present study sheds light on a novel and effective triple combination against carbapenem-resistant A. baumannii. Our *in vitro* results showed that combining imipenem with apo-transferrin and sodium ascorbate yielded synergism in 95% of test isolates, and this was associated with a marked reduction in imipenem MIC, shifting it below the breakpoint. Furthermore, a bactericidal effect was recorded, with no viable count detected at 10 h. An *in vivo* murine model of pneumonia was induced to mimic human disease. The triple combination therapy restored lung function and structure, with mild to moderate residual inflammation and moderately congested vessels observed 8 h following the initiation of therapy. Therefore, our findings suggest novel insights about a promising new combination therapy against highly resistant carbapenemase-producing A. baumannii to restore the effectiveness of imipenem.

## INTRODUCTION

The worldwide spread of bacterial pathogens capable of producing extended-spectrum β-lactamases (ESBLs) has resulted in a global rise in carbapenem consumption (1, 2). Such pressure has led to the emergence as well as the spread of carbapenemase-producing pathogens, such as Acinetobacter baumannii, which has become a major problem, particularly for immunodeficient patients, due to severe infections associated with a high rate of mortality (2, 3). Dosing regimens, including prolonged infusion of imipenem (IMP) alone, may be effective for strains recording certain MIC values (up to 8 mg/L). However, carbapenemase-producing A. baumannii isolates have higher MICs (4, 5). Therefore, it is necessary to find adjuvant or alternative modalities to treat severe infections caused by these isolates.

Vitamin C is considered an inexpensive and available adjuvant with few or no side effects (6). It is commonly prescribed as a dietary supplement. Additionally, it is characterized by its antioxidant properties and has been added as an adjuvant in protocols of cancer chemotherapy (7, 8). Several studies have reported that ascorbic acid has significant antibacterial activity against bacterial pathogens such as Corynebacterium diphtheriae, Staphylococcus aureus, Helicobacter pylori, and Mycobacterium tuberculosis (9–11). Moreover, ascorbic acid enhanced the antibiofilm effect of levofloxacin on urethral catheters (12). Furthermore, it was reported that vitamin C salts like copper ascorbate showed an inhibitory effect on Serratia marcescens. Additionally, combining sodium ascorbate (ASCO) with ciprofloxacin had certain synergistic activity against biofilm-forming Pseudomonas aeruginosa (13).

Most microbial pathogens need exogenous sources of iron for survival and growth, and consequently, iron is essential for host colonization establishment as well as infection initiation (14–16). Numerous studies have described some small iron-sequestering molecules that are capable of inhibiting microbial growth by reducing the availability of iron for the pathogen and hence preventing the development of infection (16–19). In particular, apo-transferrin (APO) has been reported to inhibit the growth of Candida albicans, A. baumannii, and Staphylococcus aureus. Additionally, transferrin and lactoferrin could inhibit biofilm formation by P. aeruginosa. Lin et al. (20) stated that apo-transferrin showed a synergistic effect with rifampicin against S. aureus for which an 8-fold reduction in the MIC of rifampicin was recorded.

There are no available data about the use of apo-transferrin and ASCO combined with IMP against carbapenemase-producing A. baumannii. So the present study focused on investigating the *in vitro* as well as the *in vivo* efficacy of this triple combination as a rescue therapeutic possibility in a mouse pneumonia model of multidrug-resistant (MDR) A. baumannii.

## RESULTS

Table 1 presents variants of carbapenemases and multilocus sequence type (MLST) clones of 20 A. baumannii test isolates previously determined by Al-Hassan and Al-Madboly (21). Genotypic analysis revealed that isolates harbored one or more variants of carbapenemases, including OXA-23, OXA-66, OXA-69, OXA-86, OXA-94, NDM-1, and/or VIM-2, resulting in various resistance profiles determined based on the 2019 European Antimicrobial Susceptibility Testing Committee (EUCAST) breakpoints as recorded in Table 1.

**TABLE 1:** Resistance profile of test A. baumannii isolates, sequence type, and molecular analyses of β-lactamases

Strain	Resistance profile^*a*^	Sequence type (MLST)	Variant(s) of carbapenemases
Ac 30	AMP-AMP/SUL-PIP/TZB-FEP-IMP-MEM-AMK-GEN-CIP-LVX-SXT	ST499	NDM-1
Ac 42	AMP-AMP/SUL-PIP/TZB-FEP-IMP-MEM-AMK-GEN-CIP-LVX-SXT	ST1294	NDM1, OXA-23, OXA-66
Ac 91	AMP-AMP/SUL-PIP/TZB-FEP-IMP-MEM-AMK-GEN-CIP-LVX-SXT	ST1078	NDM-1, OXA-94
Ac 237	AMP-AMP/SUL-PIP/TZB-FEP-IMP-MEM-SXT	ST1078	NDM-1, OXA -94
Ac 139	AMP-AMP/SUL-PIP/TZB-FEP-IMP-MEM-AMK-GEN-CIP-LVX-SXT	ST1298	NDM-1, OXA-66
Ac 136	AMP-AMP/SUL-PIP/TZB-FEP-IMP-MEM-AMK-GEN-SXT	ST1296	VIM-2, OXA-66
Ac 62	AMP-AMP/SUL-PIP/TZB-FEP-IMP-MEM-AMK-GEN-SXT	ST1289	NDM-1, OXA-23, OXA-66
Ac 83	AMP-AMP/SUL-PIP/TZB-FEP-IMP-MEM-SXT	ST1078	NDM-1, OXA23, OXA-94
Ac 26	AMP-AMP/SUL-PIP/TZB-FEP-IMP-MEM-AMK-GEN-CIP-LVX-SXT	ST231	OXA-23, OXA-69
Ac 20	AMP-AMP/SUL-PIP/TZB-FEP-IMP-MEM-AMK-GEN-CIP-LVX-SXT	ST391	OXA-68
Ac 22	AMP-AMP/SUL-PIP/TZB-FEP-IMP-MEM-AMK-GEN-CIP-LVX-SXT	ST1078	NDM-1, OXA-94
Ac 41	AMP-AMP/SUL-PIP/TZB-FEP-IMP-MEM-AMK-GEN-CIP-LVX-SXT	ST1293	VIM-2, OXA-23, OXA-66
Ac 50	AMP-AMP/SUL-PIP/TZB-FEP-IMP-MEM-AMK-GEN-CIP-LVX-SXT	ST1289	OXA-23, OXA-66
Ac 8	AMP-AMP/SUL-PIP/TZB-FEP-IMP-MEM-AMK-GEN-CIP-LVX-SXT	ST1289	OXA-23, OXA-66
Ac 44	AMP-AMP/SUL-PIP/TZB-FEP-IMP-MEM-AMK-GEN-CIP-LVX-SXT	ST1295	OXA-23, OXA-66
Ac 15′	AMP-AMP/SUL-PIP/TZB-FEP-IMP-MEM-SXT	ST195	OXA-23, OXA-66
Ac 25	AMP-AMP/SUL-PIP/TZB-FEP-IMP-MEM-SXT	ST1296	NDM-1, OXA-66
Ac 43	AMP-AMP/SUL-PIP/TZB-FEP-IMP-MEM-SXT	ST368	NDM-1, OXA-23, OXA-66
Ac 32	AMP-AMP/SUL-PIP/TZB-FEP-IMP-MEM-SXT	ST441	NDM-1, OXA-69
Ac 33	AMP-AMP/SUL-PIP/TZB-FEP-IMP-MEM-AMK-GEN-CIP-LVX-SXT	ST441	NDM-1, OXA-69

a AMP, ampicillin; AMP/SUL, ampicillin-sulbactam; PIP/TZB, piperacillin-tazobactam; FEP, cefepime; IMP, imipenem; MEM, meropenem; AMK, amikacin; GEN, gentamicin; CIP, ciprofloxacin; LVX, levofloxacin; SXT, trimethoprim-sulfamethoxazole.

Susceptibility of A. baumannii clinical isolates to imipenem, sodium ascorbate, and apo-transferrin was determined. Test isolates demonstrated a broader range of MIC values (8 to 128 μg/mL) when susceptibility to imipenem was assessed (Table 1). In total, all test isolates (100%) were imipenem resistant (MIC of imipenem [MIC_imp_] > 4 mg/L). However, high MIC values were recorded for sodium ascorbate (512 to 1,024 μg/mL) compared to apo-transferrin (64 to 256 μg/mL), as presented in Table 2.

**TABLE 2:** Susceptibility testing of A. baumannii isolates for single drugs and the triple combination, as well as synergism testing results

Strain	MIC (μg/mL)^*a*^						FICI^*c*^
	Single drugs			Triple combination			
	IMP^*b*^	ASC	APO	IMP-COM	ASC-COM	APO-COM	
Ac 30	128	1,024	256	1	16	64	0.328125
Ac 42	128	1,024	256	1	256	64	0.5625
Ac 91	16	512	256	2	64	32	0.632813
Ac 237	32	512	64	0.25	64	64	0.640625
Ac 139	32	512	128	1	128	8	0.40625
Ac 36	64	1,024	64	0.5	64	16	0.320313
Ac 62	16	1,024	64	0.5	64	64	0.59375
Ac 83	64	512	128	1	64	16	0.375
Ac 26	32	1,024	128	1	64	64	0.578125
Ac 20	8	1,024	128	1	128	16	0.376953
Ac 22	64	1,024	64	0.25	256	32	0.390625
Ac 41	128	512	256	1	128	32	0.515625
Ac 50	8	1,024	128	2	64	64	0.8125
Ac 8	128	512	128	1	16	32	0.546875
Ac 44	64	1,024	64	0.5	128	32	0.515625
Ac 15′	32	512	128	0.5	16	64	0.546875
Ac 25	32	512	128	0.5	64	128	0.65625
Ac 43	32	1,024	256	1	256	8	0.378906
Ac 32	32	1,024	64	0.5	128	32	0.382813
Ac 33	128	1,024	128	1	128	32	0.4375
A. baumannii ATCC 1506	1	8	2				
P. aeruginosa ATCC 27853	1	4	4				

a IMP, imipenem; ASCO, ascorbate; APO, apo-transferrin; COM, combination.

b Imipenem breakpoint is >4 mg/L according to EUCAST 2019.

c FICI, fractional inhibitory concentration index.

The results of the checkerboard assay revealed indifference or no interaction (0.8 < fractional inhibitory concentration index [FICI] < 4) showed by double combinations (IMP-ASCO, IMP-APO, and ASCO-APO), while a synergistic effect (FICI < 0.8) was detected in 95% of test isolates after treatment with a triple combination of imipenem with apo-transferrin and sodium ascorbate as recorded in Tables 2 and 3. Moreover, MIC_imp_ was strongly reduced in triple combination for highly resistant isolates (up to 128-fold reduction). Interestingly, MIC values were decreased below the EUCAST breakpoint for imipenem (<4 mg/L). Additionally, an absence of significant correlation (ρ = −0.61; *P = *0.15) between MIC_imp_, FICI, and the carried β-lactamase variants was observed, indicating the absence of a causal relationship between the recorded synergism with imipenem and the β-lactamase type. Interestingly, supplementation of medium with metals (FeCl_3_ or ZnCl_2_) or hemin at different concentrations did not show a significant effect on the results of triple combination as recorded in Table 4.

**TABLE 3:** Susceptibility testing for double combinations, as well as the synergism testing

Strain	IMP/ASC			IMP/APO			ASC/APO		
	MIC (μg/mL)		FICI	MIC (μg/mL)		FICI	MIC (μg/mL)		FICI
	IMP-COM	ASC-COM		IMP-COM	APO-COM		APO-COM	ASC-COM	
Ac 30	64	512	1	64	128	1	256	512	1.5
Ac 42	128	128	1.125	64	128	1	256	1,024	2
Ac 91	16	64	1.125	8	128	1	128	512	1.5
Ac 237	32	32	1.0625	16	32	1	64	256	1.5
Ac 139	8	512	1.25	8	32	1	32	512	1.25
Ac 36	32	512	1	16	32	1	32	512	1
Ac 62	16	128	1.125	16	16	1.125	32	1,024	1.5
Ac 83	64	64	1.125	32	64	1	64	256	1
Ac 26	32	64	1.0625	32	64	1	128	256	1.25
Ac 20	4	256	0.75	4	32	1	128	512	1.5
Ac 22	32	256	0.75	32	32	1	64	512	1.5
Ac 41	64	256	1	64	128	1	256	256	1.5
Ac 50	64	128	1.125	32	64	1	128	512	1.5
Ac 8	64	128	0.75	64	32	0.75	64	128	1
Ac 44	64	256	1.25	32	32	1	64	1,024	2
Ac 15′	32	32	1.0625	16	64	1	32	256	1
Ac 25	32	64	1.125	16	64	1	128	128	1.25
Ac 43	32	128	1.125	32	32	1.125	256	512	1.5
Ac 32	16	512	1	16	64	1	16	1,024	1.25
Ac 33	64	512	1	64	64	1	64	1,024	1.5

**TABLE 4:** Effect of metal or hemin supplementation on the triple combination activity against test isolates^*a*^

Strain	Effect of:					
	Treatment with 8 μg/mL of apo-transferrin			Treatment with 32 μg/mL of apo-transferrin		
	FeCl_3_	ZnCl_2_	Hemin	FeCl_3_	ZnCl_2_	Hemin
Ac 30	−	−	−	−	−	−
Ac 42	−	−	−	−	−	−
Ac 91	−	−	−	−	−	−
Ac 237	−	+	−	−	−	−
Ac 139	−	−	−	−	−	−
Ac 136	−	−	−	−	−	−
Ac 62	−	−	+	−	−	−
Ac 83	−	−	−	−	−	−
Ac 26	+	−	−	−	−	−
67 20	−	−	−	−	−	−
Ac 22	−	−	−	−	−	−
Ac 41	−	−	−	−	−	−
Ac 50	−	−	++	−	−	+
Ac 8	−	−	−	−	−	−
Ac 44	−	−	−	−	−	−
Ac 15′	−	−	−	−	−	−
Ac 25	−	−	−	−	−	−
Ac 43	−	+	−	−	−	−
Ac 32	−	−	−	−	−	−
Ac 33	−	−	−	−	−	−

a −, no reverse effect detected; +, reverse effect detected at 10× apo-transferrin MIC; ++, reverse effect observed at both 1× and 10× apo-transferrin MIC. There was a nonsignificant increase in MIC values that was still in the susceptible range.

**FIG 1:**
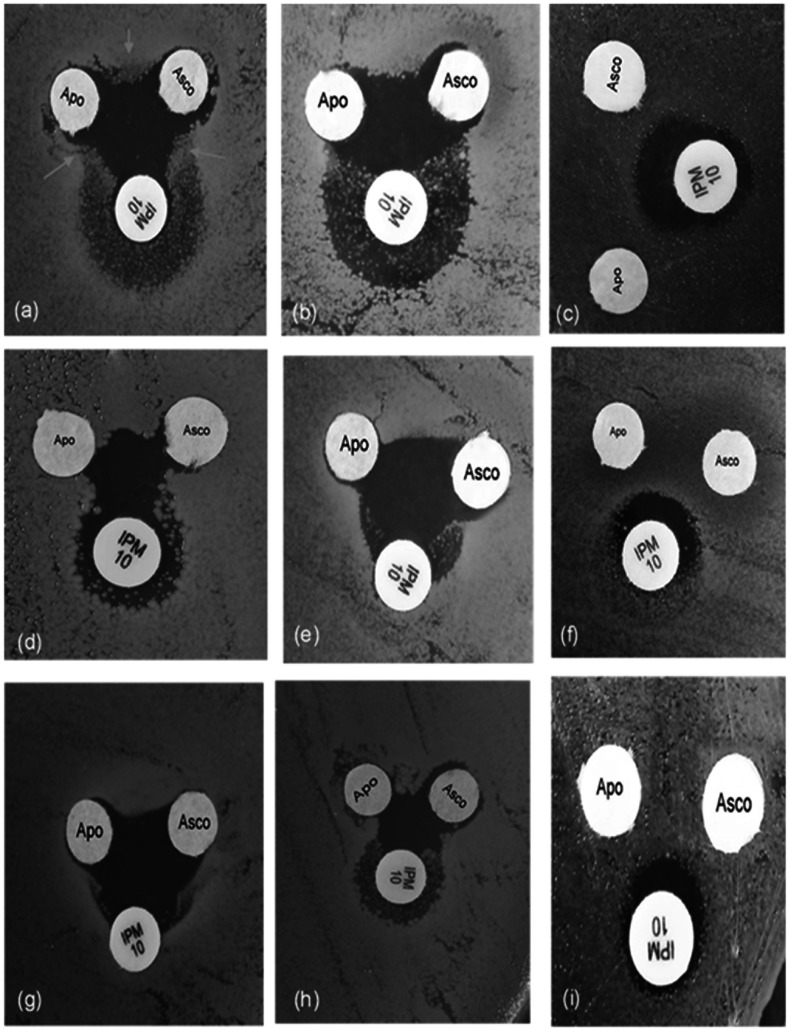
Disc diffusion test showing inhibition zones of imipenem, ascorbate, and apo-transferrin of representative A. baumannii isolates: Ac 136 (a), Ac 20 (b), Ac 33 (c), Ac 8 (d), Ac 30 (e), Ac 36 (f), Ac 42 (g), Ac 83 (h), and Ac 139 (i). Clear synergism was detected as common inhibition zones at the centers of the three test agents. White arrows point to the growth present between the two-test-drug-containing discs, which means absence of synergism between double combinations.

To support the data of the checkerboard assay, a disc diffusion test was carried out. Nine isolates were selected to represent the synergistic effect. The results revealed a synergistic pattern for all selected isolates similar to that of the checkerboard assay (Fig. 1). Additionally, the synergy of imipenem with both apo-transferrin and sodium ascorbate was also visible for strains that showed growth of mutants inside the inhibition zone of imipenem, which was inhibited by the synergistic activity of other test agents (Fig. 1). For double combinations, the growth detected between the two-test-drug-containing discs means the absence of synergism between double combinations, as pointed out by the arrows in Fig. 1.

**FIG 2:**
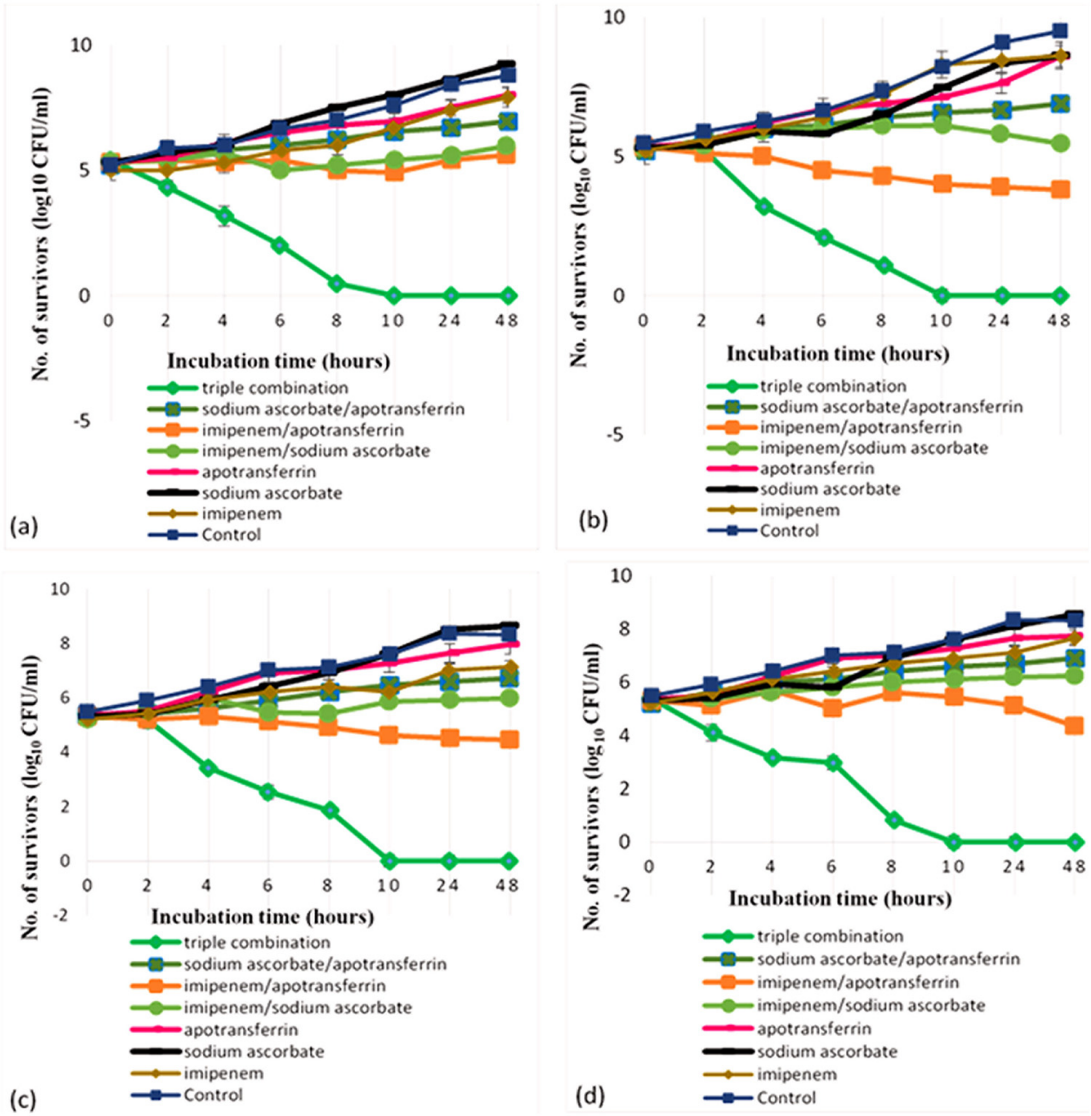
Assessment of the triple combination of imipenem (4 μg/mL), 1/4 MIC of both apo-transferrin, and sodium ascorbate against A. baumannii representative test isolates—Ac30 (a), Ac36 (b), Ac42 (c), and Ac83 (d)—using a time-kill assay, showing zero viable count after 10 h of incubation with the triple combination.

Figure 2 presents the results of a time-kill assay of monotherapy as well as double and triple combinations. All test monotherapies showed nonsignificant reduction. Furthermore, double combinations showed little but also a nonsignificant decrease in the CFU after a long contact time. For example, the combination of imipenem and apo-transferrin yielded only a 1-log_10_ reduction in the viable count after 8 h of incubation, and this decrease reached 1.5 log_10_ following 48 h (Fig. 2b). Triple combinations of imipenem with ascorbate and apo-transferrin showed synergistic effects, with 2-log_10_ reductions in CFU following 4 h of incubation compared to imipenem alone or its double combinations, as shown in Fig. 2. Furthermore, this decrease in CFU continued until reaching up to 4-log_10_ reductions at 8 h compared to the initial count recorded at zero time, indicating a bactericidal effect. Additionally, no viable count was recorded at 10 h for all test representative isolates. Interestingly, triple combination presented synergistic activities with ≥2-log_10_ decreases compared to single and double combination treatment as shown in time-kill curves presented in Fig. 2.

**FIG 3:**
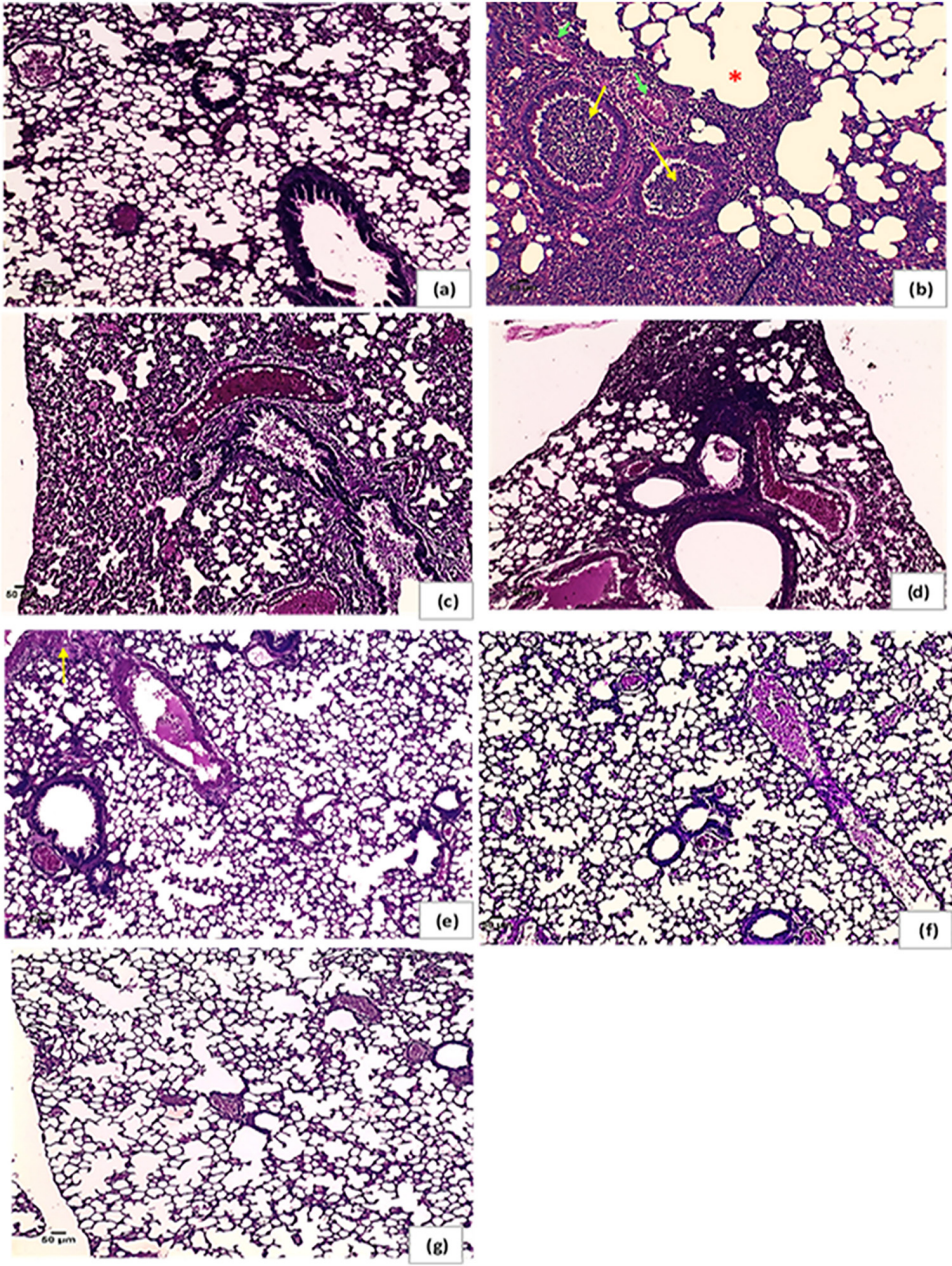
Histopathology of pulmonary tissues, H&E stained, representing different groups of the animal model. (a) Negative-control group showing normal lung with thin wall alveoli. (b) Bacterium-infected positive-control group; heavy inflammatory infiltrate was detected with intrabronchial heavy suppurative exudate (yellow arrows) and marked surrounding congested vessels (green arrows). Emphysematous changes or compensatory emphysema in alveoli was seen (asterisk). (c) Imipenem-treated group showing failure of therapy represented in dilated bronchioles filled with inflammatory exudate, markedly congested blood vessels, and few remaining patent alveoli. (d) A representative double combination-treated group showing absence of improvement represented by heavy cellular inflammatory infiltrate within lung parenchyma in addition to dilated congested vessels. (e) Triple-combination rescue group showing within normal lung with mild to moderate residual inflammation (arrow) and moderately congested vessels after 8 h of administration. (f) Triple-combination rescue group after 24 h of treatment showing mild residual inflammation and mildly congested vessels. (g) Triple-combination rescue group following 72 h of treatment showing normal lung with normal patent alveoli. Magnification, ×100.

**FIG 4:**
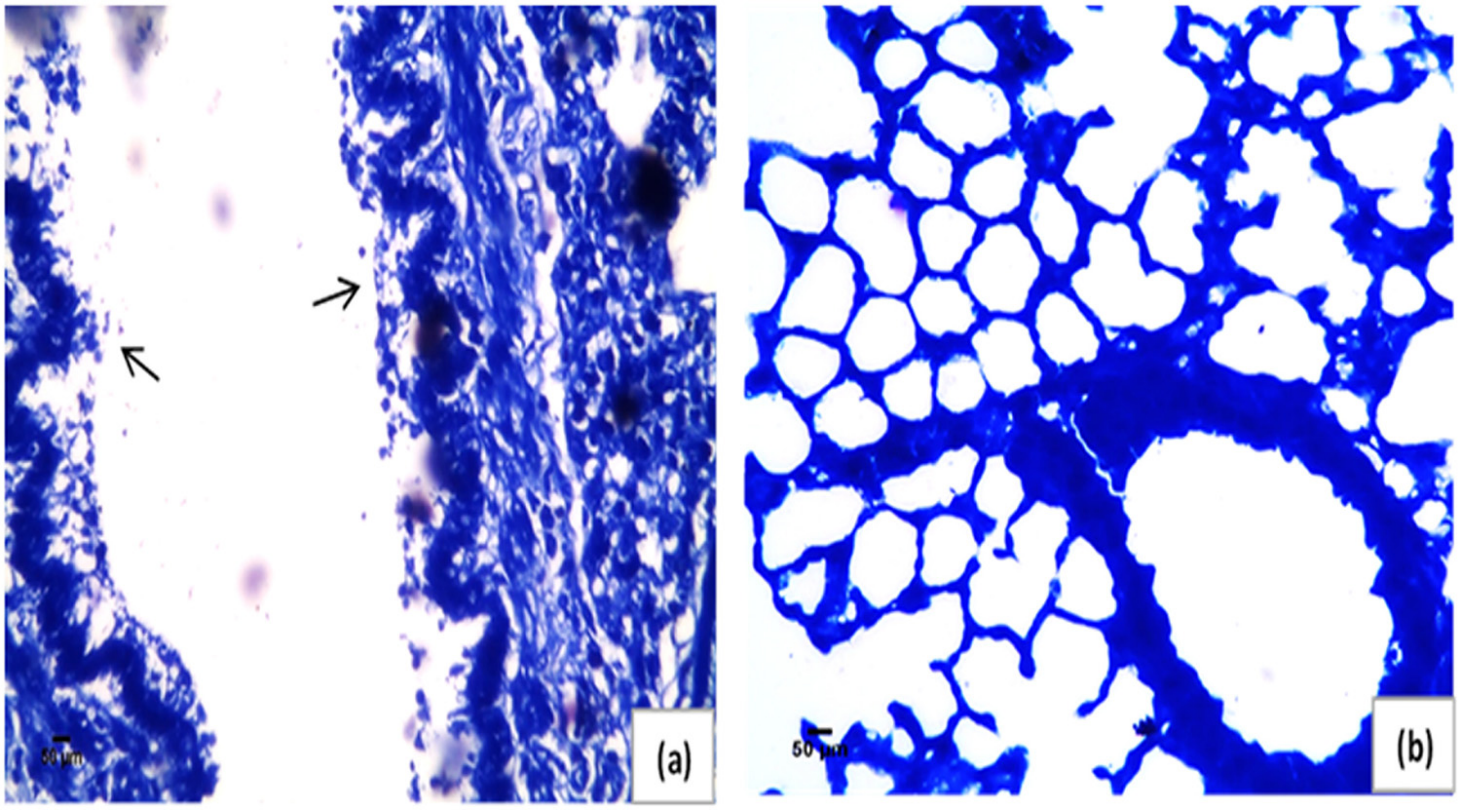
Giemsa-stained lung tissues for histopathological examination showing prebronchial heavy inflammatory infiltrate and rods (arrows) of the A. baumannii challenge strain in an infection-positive control (a) and normal lung free from bacterial cells following treatment with triple combination rescue therapy (b). Magnification, ×200.

**FIG 5:**
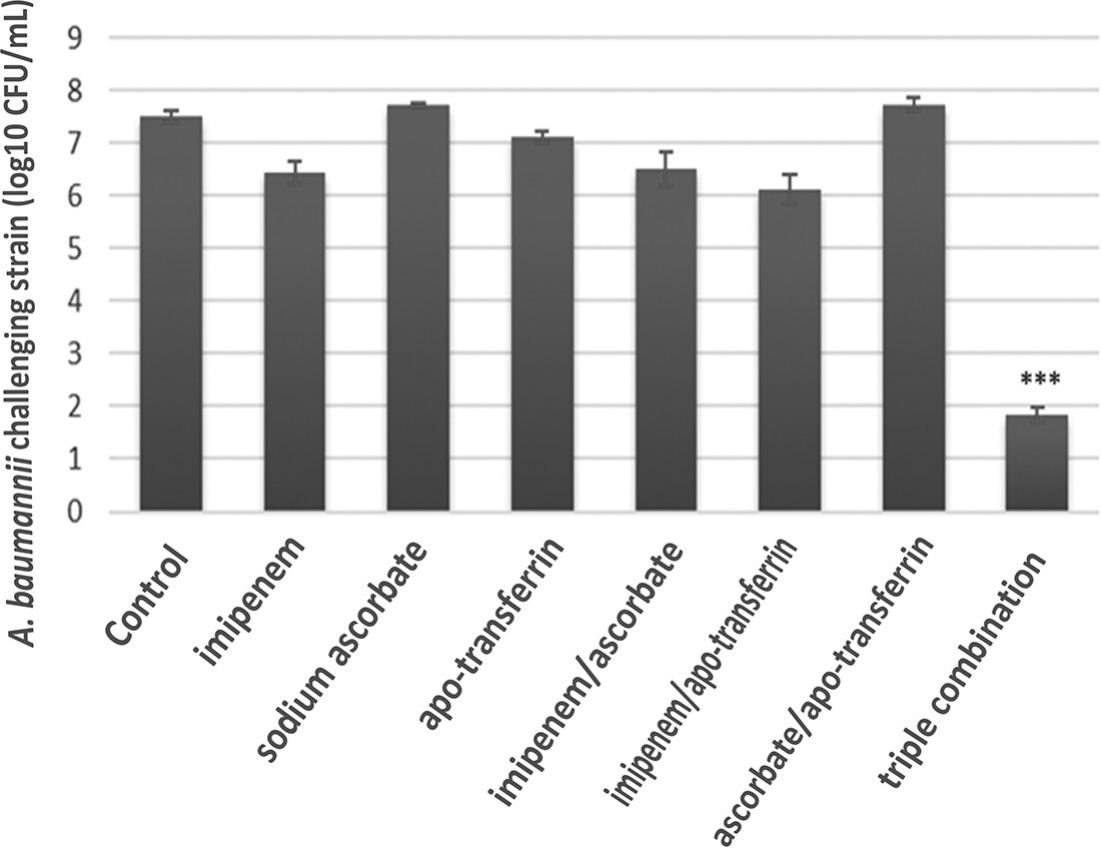
Bacterial burdens of A. baumannii challenge microorganism in lung were determined quantitatively by viable count at 24 h postinoculation. Data are presented as means ± SD, and the experiment was carried out in triplicate. Significance of results was determined when the *P* value was <0.001 (***).

**FIG 6:**
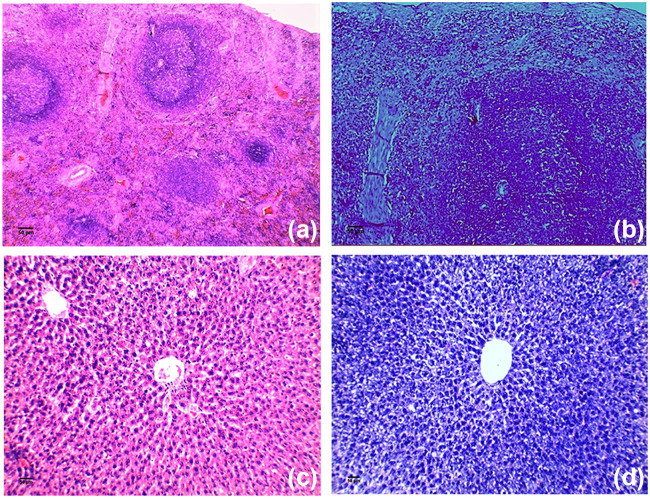
Histopathological images of spleen section stained with H&E (magnification, ×40) (a), spleen section stained with Giemsa (magnification, ×100) (b), liver section stained with H&E (magnification, ×200) (c), and liver section stained with Giemsa (magnification, ×200) (d), obtained from A. baumannii-infected mice, showing normal histology, indicating absence of bacterial dissemination outside the lungs.

Pulmonary tissue obtained from the Acinetobacter-infected positive-control group revealed heavy inflammatory infiltrate characterized by intrabronchial suppurative exudate and marked surrounding congested vessels and compensatory emphysema in alveoli Fig. 3b compared to the negative-control group (Fig. 3a). No improvement was noticed in either single or double compound therapy (Fig. 3c and d). On the other hand, the use of a triple combination resulted in restoration of the lung function and structure, with mild to moderate residual inflammation and moderately congested vessels observed 8 h following administration (Fig. 3e). Additionally, after 24 h of treatment, mild residual inflammation and mildly congested vessels were seen (Fig. 3f) and normal lungs with normal patent alveoli were detected 72 h following treatment (Fig. 3g). Also, Giemsa-stained sections showed rods of Acinetobacter in the animal group infected with the test pathogen which disappeared in the sections obtained from the triple rescue group after 72 h (Fig. 4). Bacteriological analysis showed a highly significant difference between the triple therapy group and others (*P* ≤ 0.001) as shown in Fig. 5, where 4-log_10_ reductions were recorded compared to the results for the most active single drug, indicating synergy according to the definition. Furthermore, about 5-log_10_ reductions were determined compared to the initial count at zero time, indicating bactericidal effect based on the definition. Additionally, there was no extrapulmonary dissemination detected, i.e., no bacteria were observed in the spleen or liver, as shown in the histopathological images presented in Fig. 6.

## DISCUSSION

Carbapenem-resistant A. baumannii is considered the most dangerous pathogen, causing hospital-acquired pneumonia as well as bloodstream infections leading to high rates of morbidity and mortality (3). Additionally, the limited treatment options due to development of multidrug resistance necessitate the search for new therapeutic agents or new adjuvants to form combinations (22).

To determine the best-performing therapeutic agents in the present work, *in vitro* synergy testing was carried out using a checkerboard assay. My data revealed that the triple combination of imipenem, apo-transferrin, and sodium ascorbate was more effective than monotherapy or double combination therapy regardless of the number of carbapenemase variants harbored by the test isolates. In the context of clinical application, synergism is valuable only if the MIC value is reduced below the respective breakpoint. Accordingly, the high MIC values of imipenem (up to 128 μg/mL) recorded against the test isolates were reduced below the susceptibility breakpoints after subjection to triple combination (<4 μg/mL), with up to a 128-fold reduction compared to that obtained with imipenem alone. Furthermore, the addition of apo-transferrin and sodium ascorbate to the imipenem breakpoint made the latter bactericidal for carbapenem-resistant A. baumannii isolates: 2-log_10_ reductions in CFU of strain Ac 42 were recorded after 4 h of incubation compared to the initial count recorded at zero time in the time-kill assay. This reduction was continued until no viable count was recorded at 10 h. Also, the triple combination presented synergistic activities with ≥2-log_10_ decreases in viable count compared to those with the single and double therapy regimens. Moreover, the disc diffusion test confirmed the results of the checkerboard assay regarding the triple combination that inhibited the emergence of resistant mutants. Interestingly, this work represents the first report about the potent activity of the triple combination against carbapenem-resistant A. baumannii isolates. Luna et al. (14) reported that a double combination of apo-transferrin and ciprofloxacin or meropenem resulted in an additive antimicrobial killing effect, against Klebsiella pneumoniae and A. baumannii strains, *in vitro* during dynamic time-kill assays without showing synergism or antagonism for the majority of test bacterial strains. Additionally, apo-transferrin showed antibacterial activities at concentrations that could be achieved *in vivo* through patient infusion. Moreover, administration of 1,040 mg/kg of body weight infused into transplant patients did not show any toxicities. So, serum transferrin concentration would reach 14 mg/mL (for a 70-kg patient), and this level was higher than the apo-transferrin MICs. In the presence of transferrin, the recorded MIC shift was 10- to 100-fold lower than for the antibiotic alone. On the other hand, the addition of hemin reversed the effect of apo-transferrin, and this is the alternative pathway or system expressed by K. pneumoniae and A. baumannii for iron uptake (14, 23). In the present work, the time-kill assay provided evidence of the triple combination bactericidal effect against the representative test isolates and hence synergism as recorded by checkerboard test, in which no viable cells were recorded after 10 h of incubation. Interestingly, supplementation of medium with metals (FeCl_3_ or ZnCl_2_) or hemin at different concentrations did not show a significant effect on the results of the triple combination.

The dominantly observed synergism achieved by triple combination might be explained as follows. Apo-transferrin is known to be an iron-sequestering agent that is capable of disrupting membrane potential, enhancing the antimicrobial effects and decreasing the emergence of antimicrobial resistance, so it could be used as an adjuvant to treat bacterial infections (14, 20). Additionally, iron also induces antibiotic-mediated free-radical damage of bacterial DNA through the Fenton reaction (24). Furthermore, apo-transferrin is characterized by its ability to inhibit bacterial adhesion to biomaterials (25). It was also reported that ascorbic acid and ascorbates were capable of increasing bacterial cellular permeability, leading to enhancement in the uptake of antibiotics like meropenem via the outer membrane porins of K. pneumoniae (2). This supports the relationship between the permeability of the cell membrane and wall as well as the restored antibacterial efficacy. Moreover, ascorbic acid and ascorbates are stated in the literature to be antiplasmid compounds or plasmid-curing agents and so can enhance the susceptibility of bacterial pathogens to antibiotics while reducing antimicrobial resistance development (26). Furthermore, they are stated to be quorum-sensing inhibitors which are able to affect the virulence of different pathogens (27, 28).

A. baumannii is known to be a difficult-to-treat bacterial pathogen (29). Therefore, the acute A. baumannii pneumonia model in mice was used in this study to induce bacterial pathogenesis and then evaluate the therapeutic efficacy of the triple combination. This model is characterized by a reproducible acute course of pneumonia, particularly in immunocompetent common strains of mice, whether female or male (30). The challenging clinical isolate used in this model was highly virulent when inoculated into immunocompetent mice. Results revealed a high mortality rate (100%) in the positive-control group within 48 h postinfection but without extrapulmonary dissemination; i.e., no bacteria were detected in the spleen or liver. This was in agreement with the findings of Harris et al. (30). According to the study of Hua et al. (29), pulmonary parenchyma interstitial inflammation is considered an early bacterial pneumonia stage that could be detected 2 h following intratracheal inoculation. In my work, the triple therapy was administered 3 h post-bacterial challenge to evaluate the *in vivo* therapeutic efficacy. Significant reduction in bacterial burden (4-log_10_ reduction) in pulmonary tissues was recorded 24 h after administration of triple therapy. Meanwhile, histopathological examination presented amelioration in the infected lung with a marked reduction in congestion and infiltrated inflammatory cells after triple therapy compared to double or monotherapy. Furthermore, normal lung with normal patent alveoli was restored 72 h following treatment, with the absence of Acinetobacter rods in Giemsa-stained sections.

In conclusion, data obtained in this study showed that triple combination was effective against carbapenem-resistant A. baumannii isolates compared to double combinations. Moreover, the high MICs of imipenem were shifted below the susceptibility breakpoints, indicating that strong synergistic effects resulted from the addition of apo-transferrin and sodium ascorbate, hence reducing the emergence of antibiotic resistance. Additionally, the *in vivo* triple efficacy achieved upon testing in the mouse pneumonia model was promising, showing evidence of an *in vivo* bactericidal effect.

## MATERIALS AND METHODS

### Reference strains.

Pseudomonas aeruginosa ATCC 27853 and A. baumannii ATCC 1506 were used for *in vitro* studies as quality control strains.

### Test microorganisms.

A total of 20 Acinetobacter baumannii clinical isolates were previously isolated and identified at the molecular level. Moreover, antimicrobial susceptibility patterns and variants of β-lactamases were also previously determined (21) and are presented in Table 1.

### Challenging microorganism.

Clinical isolate A. baumannii Ac 42 was selected for the *in vivo* evaluation test. It is a multidrug-resistant isolate that belongs to the ST1294 clone. It contains genes coding for three known carbapenemases of classes B and D (Table 1), as previously determined by PCR using specific primers as well as sequencing (21).

### Antibacterial agents.

Imipenem was bought from Sigma-Aldrich (USA) and was freshly prepared before each experiment. A stock solution of 5 mg/mL was prepared in RPMI 1640 medium (Lonza, USA), divided into aliquots, and stored at –20°C.

Sodium l-ascorbate was purchased from Sigma-Aldrich (USA) and was also prepared as a fresh stock solution of 5 mg/mL of OmniTrace Ultra Water (EMD Millipore Corporation).

Apo-transferrin (iron depleted) was purchased from Sigma-Aldrich (USA). It was also prepared fresh before each experiment in the form of stock solution (5 mg/mL) prepared in OmniTrace Ultra Water (EMD Millipore Corporation).

### Mice.

Female mice (C57BL/6N), specific pathogen free, with an age range between 6 and 8 weeks and weight of 16 to 18 g, were used. They were purchased from the Laboratory Animal Center of Helwan University, Egypt. Maintenance and use of animals were done in accordance with the animal care and user guide recommendations.

### Ethics statement.

The animal care committee of the Faculty of Pharmacy, Tanta University, Egypt, reviewed the animal infection protocol and approved the experimental procedures (approval number REC-TP/M0001).

### Antibacterial susceptibility testing.

**(i) Inoculum preparation.** Overnight cultures of the test isolates were grown in tryptic soy broth (TSB) that was subjected to 1:100 dilution in RPMI 1640 medium. These were subcultured until reaching an optical density at 600 nm (OD_600_) of 0.5. Bacterial cells were pelleted by centrifugation at 3,500 × *g* for 5 min. The pellet was washed three times using RPMI 1640 medium, followed by resuspension in this medium and dilution to 1 × 10^6^ CFU/mL. The density of the bacterial cells was confirmed through plating serial dilutions using Tryptic soy agar (TSA) and extrapolation of CFU per plate to CFU per milliliter (2).

**(ii) MIC determination.** The MIC values were determined according to the guidelines of the European Antimicrobial Susceptibility Testing Committee (EUCAST; version 9.0) (31). Some modifications were made, including the use of RPMI 1640 medium instead of Mueller-Hinton (MH) broth due to low iron content in the basal formulation of RPMI 1640 medium, which better represents the environment of a human host. After preparation of the inoculum, the MIC values were determined using a U-shaped, round-bottom 96-well microtiter plate. Serial 2-fold dilutions were conducted in columns across the microtiter plate to determine single-drug susceptibility. Medium-only and bacterium-alone wells were used as negative and positive controls, respectively. Bacterial cells were inoculated by transferring 100 μL/well of 1 × 10^6^ CFU/mL that was diluted to 1 × 10^5^ CFU/well. Microtiter plates were incubated at 35 ± 1°C for 18 ± 2 h. The MIC value was defined as the lowest concentration of the test compound that showed a ≥90% reduction in OD_600_ compared to that of the control (2).

### Checkerboard synergy test.

For synergy testing, a standard broth microdilution checkerboard assay was followed for double combinations; however, a three-dimensional (3D) checkerboard assay was used for the assessment of the triple combination. Briefly, the method of broth microdilution was modified by adding some additional antibiotics/drug concentrations (2). For each antibiotic and strain, the selected concentration ranges were based on a previous determination of MICs and the steps were previously described in detail. Additionally, imipenem was diluted in 11 dilution steps, while sodium ascorbate was tested in 7 dilution steps, and 6 dilution steps were investigated for apo-transferrin. Imipenem was prepared in *x* axes, sodium ascorbate was prepared in *y* axes, and apo-transferrin was added in the *z* axes as fixed sub-MICs (1/8, 1/4, or 1/2 MIC value). Plates were incubated at 35 ± 1°C for 18 ± 2 h. Each test was repeated at least thrice. For testing double (equation 1) or triple (equation 2) drug combinations, the fractional inhibitory concentration index (FICI) was calculated as follows:

(1) $${\text{FICI}}_{A/B}={\text{MIC}}_{A}\left(\text{combination}\right)/{\text{MIC}}_{A}\left(\text{alone}\right)\;+\;{\text{MIC}}_{B}\left(\text{combination}\right)/{\text{MIC}}_{B}\left(\text{alone}\right)$$

(2) $${\text{FICI}}_{A/B/C}={\text{ MIC}}_{A}\left(\text{combination}\right)/{\text{MIC}}_{A}\left(\text{alone}\right)\;+\;{\text{MIC}}_{B}\left(\text{combination}\right)/{\text{MIC}}_{B}\left(\text{alone}\right)+{\text{ MIC}}_{C}\left(\text{combination}\right)/{\text{MIC}}_{C}\left(\text{alone}\right)$$

The FICI values were calculated depending on the concentrations present in the first nonturbid well for each row and column and presented as medians. Additionally, the median averaged FICIs were used instead of the lowest value to exclude the overinterpretation of synergy due to experimental error of dilution. Therefore, FICI interpretation was done according to the methods of Odds (32) and Zhou et al. (33) for double combinations where FICI ≤ 0.5 represents synergism; 0.5 < FICI < 4 represents indifference or no interactions, and FICI > 4 refers to antagonism. For the triple combination, interpretation was carried out according to the method of Den Hollander et al. (34); FICI < 0.8 means synergy, 0.8 < FICI < 4 represents indifference or additive effects, and FICI ≥ 4 refers to antagonism.

Reversal of apo-transferrin activity by metal supplementation was tested according to the method of Luna et al. (14).

### Triple-disc diffusion test.

The triple-disc diffusion test was carried out according to EUCAST rules for testing antimicrobial susceptibility. About 0.5 McFarland of the bacterial suspension was distributed along the surface of cation-adjusted MH (CAMH) agar plates using a cotton swab. A disc of imipenem (10 μg; Oxoid) was transferred to each plate. About 10 μL of the test drug solution containing 1/4 MIC was added to sterile cellulose discs with 6-mm diameters. These discs were applied at different distances to test the single and combined effects of the drugs on bacterial growth. All plates were incubated at 35 ± 1°C for 18 ± 2 h. The extended edge of the inhibition zone for one drug toward the disc of another drug is defined as synergism (2, 31).

### Time-kill assay.

The time-kill assay was done according to the methods of Pillai et al. (35), Ku et al. (36), Zhou et al. (33), and Nwabor et al. (37), with some modifications. It was carried out using the breakpoint of imipenem (4 μg/mL) and subinhibitory concentrations (1/4 MIC) of apo-transferrin and ascorbate to evaluate the bactericidal effect of the triple combination against the test isolates. Four isolates were selected for this test: Ac 30 (NDM-1), Ac 36 (VIM-2 and OXA-66), Ac 42 (NDM-1, OXA-23, and OXA-66), and Ac 83 (NDM-1, OXA23, and OXA-94). An overnight culture of each test isolate in MH broth was used to inoculate RPMI 1640 medium containing the test compounds alone or in combinations to get a final concentration of 1 × 10^6^ CFU/mL and then incubated in a shaking incubator (100 rpm) at 37°C. Aliquots (100 μL) were withdrawn at different time points: 0, 2, 4, 6, 8, 10, 24, and 48 h. Viable count determination was performed through 10-fold dilutions for each aliquot, using 0.9% (wt/vol) NaCl solution, and then aliquots were inoculated onto MH agar and A. baumannii colonies were counted after 24 h of incubation at 35 ± 1°C. Bactericidal effect was defined as a ≥3-log_10_ reduction compared to the initial count at zero time. For synergy, it was defined as a ≥2-log_10_ reduction with drug combinations compared to the values for the most active single drug. Experiments were carried out in triplicate on separate days. Means and standard deviations were calculated, and data presented as viable survivors (log_10_ CFU per milliliter) against time (hours).

### Mouse model.

First, we made animals transiently neutropenic using intraperitoneal injection of cyclophosphamide (150 mg/kg of body weight). About 0.2 mL of the drug was injected on days 4 and 3 before the inoculation of challenge strain Ac 42 (day 0). Animals were anesthetized using 0.2 mL of 0.65% sodium phenobarbital via intraperitoneal injection before inoculation of the challenge microorganism. Intratracheal instillation through the mouth was used for infecting the animals. Briefly, 50 μL of the test microorganism at 10^8^ CFU/mL (controlled spectrophotometrically and confirmed quantitatively by culture) was instilled through a cannulated trachea with a blunt needle. Mice were kept in a vertical position for about 4 min, and then they were maintained in a 30° decubitus position until they regained consciousness. The efficacy of the inoculation was evaluated by counting the viable microorganisms in lungs excised from 2 control infected mice (just after bacterial inoculation at zero time and 3 h later). Also, to confirm pneumonia, a necropsy was carried out at 24, 48, and 72 h following inoculation.

For each experiment, animals were divided into 9 groups (*n* = 15 each). Two groups represented the control; group I represented the positive-control group of A. baumannii infection, and group II represented the negative control. Groups III to IX were infected first and then subjected to different treatments as follows: group III represented the infected animals treated with an intraperitoneal injection of 100 mg/kg of imipenem (0.2 mL), group IV was injected with 0.2 mL of sodium ascorbate (256 mg/kg), group V was injected with apo-transferrin (64 mg/kg), group VI was treated with the double combination of imipenem and sodium ascorbate acid, group VII was treated with imipenem and apo-transferrin, group VIII was treated with sodium ascorbate and apo-transferrin, and, finally, group IX was injected with the triple combination of imipenem, apo-transferrin, and sodium ascorbate. The treatment protocol was initiated 3 h after inoculation, and drugs were given by intraperitoneal injection as single daily doses.

Animals were weighed daily and examined for weight changes; mortality was examined until day 7. Circulating neutrophil count was monitored using a hemocytometer. Lungs were excised for histopathological and bacteriological studies (30, 36).

### Histopathological examination.

Following excision, the lungs were immersed in a fixative solution known as Bouin’s fixative until examination. Fixed lung slices about 5 mm thick were dehydrated within graded alcohol solution and then embedded in paraffin, and thin sections (6 μm) were cut. Hematoxylin and eosin (H&E) and Giemsa dyes were used to stain mounted sections to facilitate light microscopy of bacterial rods (30, 38).

### Bacteriological analysis.

Collection of lungs was carried out daily from day 1 until day 7 for all groups. Lungs were excised, weighed, and then subjected to homogenization in 10 mL of normal saline. Plating of serially 10-fold-diluted homogenates was performed using tryptone soy agar. Data were expressed as mean values of log_10_ CFU per gram of lung tissues, and the standard deviation (SD) was also calculated. Bactericidal effect was defined as a ≥3-log_10_ reduction compared to the initial count at zero time. For synergy, it was defined as a ≥2-log_10_ reduction with drug combinations compared to the values for the most active single drug. Experiments were carried out in triplicate.

### Statistical analyses.

Viable bacterial counts were calculated as the means ± SD. Differences between various treatments were determined using one-way analysis of variance (ANOVA). Intergroup differences in the bacterial counts were analyzed by independent *t* test. Differences were considered to be statistically significant in all tests when the *P* value was <0.05. Spearman’s rank correlation coefficients were determined (95% confidence intervals [CIs]) for MIC variables with FICIs and β-lactamase variants using SPSS (version 16).

### Data availability.

The raw data supporting the conclusions of this article will be made available, without undue reservation, to any qualified researcher.
